# Cytotoxic Activity of Bioactive Compound 1, 2- Benzene Dicarboxylic Acid, Mono 2- Ethylhexyl Ester Extracted from a Marine Derived Streptomyces sp. VITSJK8

**Published:** 2014

**Authors:** Kannabiran Krishnan, Abirami Mani, Subashini Jasmine

**Affiliations:** *School of Biosciences and Technology, VIT University, Tamilnadu, India.*

**Keywords:** *Streptomyces *sp. VITSJK8, 1, 2- benzene dicarboxylic acid, mono 2- ethylhexyl ester, cytotoxicity, MTT assay

## Abstract

Marine *Streptomyces* are prolific producers of majority of bioactive secondary metabolites which are used in pharmaceutical industry as effective drugs against life threatening diseases. The cytotoxic activity of the pure compound 1, 2- benzene dicarboxylic acid, mono 2- ethylhexyl ester (DMEHE) from marine derived actinomycete* Streptomyces *sp. VITSJK8 was investigated against mouse embryonic fibroblast (NIH 3T3) and human keratinocyte (HaCaT) normal cell lines, human hepatocellular liver carcinoma (HepG 2) and human breast adenocarcinoma (MCF-7) cell lines by using MTT assay. The compound DMEHE exhibited IC 50 values of 42, 100, 250 and 500 µg/ ml against HepG2, MCF-7, HaCaT and NIH 3T3 cell lines, respectively. The effect of DMEHE on the growth of cancer cell lines was expressed as the % of viability. Cell viability was recorded as 67.7%, 78.14%, 82.23% and 96. 11% in HepG2, MCF-7, HaCaT and NIH 3T3 cells, respectively. The results of the study conclude that the bioactive compound isolated from the potential isolate *Streptomyces *sp. VITSJK8 exhibited cytotoxic activity against HepG2 and MCF- 7 cancer cell lines and low toxicity against normal HaCaT and NIH 3T3 cell lines.

Marine microorganisms continue to be a potential source of bioactive secondary metabolites. 70% of earth surface is covered with water and it has been estimated that only 1% of microorganisms has been cultured and identified (1). Marine actinomyetes are ubiquitous in marine sediments but at lower numbers than in soil (2). Marine derived actinomycetes have attracted researchers since they have unique metabolic and diverse forms of chemical compounds with potent cytotoxic activity and greater therapeutic efficiency (3). Actinomycetes produce unlimited sources of novel compounds which can be used for drug development. The actinobacteria play a very important role among the marine bacterial communities, because of its diversity and ability to produce novel chemical compounds of high commercial value (4).

*Streptomyces* are saprophytic gram positive bacteria which have the ability to produce vast number of structurally and functionally wide variety of antibiotics with clinical and industrial importance and are responsible for the production of about half of the discovered bioactive compounds (5). Antibiotics and other secondary metabolites from *Streptomyces* are tremendous and widely used in the human therapy, veterinary, agriculture, scientific research and in countless other areas (6). 45% of the bioactive metabolites available in the market at present are from actinomycetes and some of the important antibiotics are gentamycin, erythromycins, vancomycin or rifamycin and the recently introduced chemotherapeutic and agricultural agents such as ziracin, spynosin and dalbavacin (7).

Research on actinomycetes has slightly decreased and their share among all microbial products is only 30 to 35% in the last decade, in contrast to 75 to 80% earlier (8). The changes in the interest towards the favorite microbes depend on new expectations. Actinomycetes in particular, still remain the richest and the most versatile source of new antibiotics of improved efficacies. Marine actinomycetes still appear to be an important potential source of bioactive secondary metabolites for drug development in pharmaceutical industries (9). Members of actinomycetes are producers of clinically important antitumor drugs such as anthracyclines (aclarubicin, daunomycin and doxorubicin), peptides (bleomycin and actinomycin D), aureolic acids (mithramycin), enediynes (neocarzinostatin), antimetabolites (pentostatin), carzinophilin, mitomycins and others (10).

Cancer is one of the major causes of death in developed countries, together with cardiac and cerebrovascular diseases (11). More than 60% of approved antitumor drugs are derived from natural products. The compounds from *Streptomyces* sp. play a dominant role as a source of drug discovery and also for the development of conventional drugs for the treatment of infectious diseases. Cytotoxic compounds such as saptomycins, cremeomycin, clecarmycins, moromycins A and B, saquayamycin B were recorded from *Streptomyces* sp. (12, 13). A quinoline derivate methyl 8-(3-methoxy-3-methylbutyl)- 2- methylquinoline- 4- carboxylate, isolated from the culture of *Streptomyces* sp. neau50 showed cytotoxicity against human lung adenocarcinoma cell line A549 with an IC50 value of 29.3 µg/ml (14). A marine- derived actinomycete *Streptomyces xinghaiensis* NRRL B24674T produces a novel alkaloid compound Xinghaiamine A, exhibiting potent cytotoxic activity against human cancer cell lines MCF-7 and U-937 with the IC50 of 0.6 and 0.5 µM, respectively (15)**.**

*Streptomyces *sp. VITSJK8 is a potential isolate exhibiting various biological activities which include broad spectrum of activity against the ESBL strains with MIC values ranging from 0.13 to 2.00 µg/ ml**.** An active compound was isolated and purified by column and preparative – HPLC and identified as 1, 2-benzene dicarboxylic acid, mono 2-ethylhexyl ester (DMEHE) with a molecular formula of C_16_H_22_O_4 _(16). In this study, the cytotoxic activity of DMEHE on selected normal and cancer cell lines was investigated.

## Materials and Methods

**Isolation and** **characterization of the potential isolate**

Marine actinomycetes isolate used in this study were isolated from a sediment sample collected at Cheyyur beach, Bay of Bengal, Kanchipuram, India, using actinomycetes isolation agar (AIA) medium and the pure isolate of VITSJK8 was maintained on international *Streptomyces* project-1 (ISP1) medium (tryptone yeast extract) at 4 °C (17).

The morphological characteristics of the active isolate showed an abundant matured spore with pink color which was observed in ISP 1 with an incubation time of 7-9 days. The isolate VITSJK8 was able to grow at 28- 32 °C, pH 7.0 and it could tolerate 2% NaCl and produced brown pigment on ISP-7 medium. The 16S rRNA partial gene sequencing of actinomycetes isolate VITSJK8 yielded 837 nucleotides (GenBank accession number KF289838). The isolate using NCBI sequence database showed maximum (99%) similarity with *Streptomyces fradiae* (accession - JF9153041). Based on the molecular taxonomy and phylogeny, the isolate was identified as *Streptomyces* species and designated as *Streptomyces *sp. VITSJK8.

**16S rRNA secondary structure and restriction sites analysis**

The secondary structure and the restriction sites in the 16S rRNA sequence of the isolate VITSJK8 were predicted using online softwares Genebee and NEB Cutter (version 2.0) respectively.

**Extraction, purification and identification of bioactive compound**

The seed was prepared by inoculation of one mature colony of *Streptomyces *sp. VITSJK8 in 50 ml of ISP1 medium and kept in a shaker at 120 rpm for 3 days. Furthermore, the seeds were inoculated in 1l of ISP1 medium and kept in a shaker at 120 rpm for 9 days. After the fermentation period, the culture was harvested and the secondary metabolites were extracted using ethyl acetate (EA) as solvent (1:1 v/v). The solvent layer was concentrated using rotary evaporator to give crude extract (17)**.** The yield of EA extract was found to be 130 µg per1000 ml of the culture broth. The crude suspension (2 g) was chromatographed over silica gel column (60-120 mesh, SRL, India), and eluted with chloroform: methanol (98:2) at a constant flow rate. The active fractions were collected and concentrated.

**Spectroscopic analysis of the purified compound**

The UV-visible absorption spectra of the compound were determined using UV- visible spectrometer (SL, 210, ELICO, India) and λ max was measured. The key functional groups present in the compound were determined by Fourier transform infrared spectroscopy (FTIR) (Thermo Nicolet-330 USA). Gas chromatography– mass spectroscopy (GC- MS) was used by injecting (1 µl) of the sample with the injection temperature at 220 °C into the spectrometer equipped with HP-5 capillary column. Proton (1H) and carbon (13C) ^1^H and ^13^C nuclear magnetic resonance spectra (NMR) (Bruker Advance III 500 mHz A V 500) was used to exploit the atomic structure of the compound (16). The structure of the compound was established with the help of spectral data obtained from various spectroscopic techniques. The 3D structure of the compound was obtained by using ChemDraw software (Ultra 8.0).

**Analysis of cytotoxic activity of** **1, 2-Benzene dicarboxylic acid, mono (2-ethylhexyl) ester**

The compound DMEHE isolated from *Streptomyces *sp. VITSJK8 was investigated for the cytotoxic activity against NIH 3T3, HaCaT, Hep G2 and MCF 7 cells by the method of MTT (3-[4, 5-dimethylthiazol-2-yl] 2, 5-diphenyl tetrazolium bromide) assay.

The cell lines were obtained from the American type culture collection (ATCC, Rock Ville, MD). Cells were cultured in DMEM medium, supplemented with 10% fetal bovine serum and 100 mg/ l penicillin (Himedia, India). The cells were maintained under 97% humidity in a biological incubator at 37 °C in a 5% of carbon dioxide atmosphere. The cell numbers were determined with a hemocytometer and the viability was assessed using the MTT assay described earlier (18).

The principle of the assay is based on the reduction of soluble yellow tetrazolium salt to insoluble purple formazan crystals by metabolically active cells. Only live cells are able to take up the tetrazolium salt. The enzyme (mitochondrial dehyd-rogenase) present in the mitochondria of the live cells is able to convert internalized tetrazolium salt to formazan crystals, which are purple in colour. Then the cells were lysed and dissolved in DMSO solution. The intensity of the color developed was measured using ELISA reader (Bio- Rad 680, India) at 570 nm. 

For the MTT assay the NIH 3T3, HepG2, HaCaT and MCF-7 cells were plated separately in 96 well plates at a concentration of 1×10^5^ cells/ well. After 24 h incubation, cells were washed twice with 100 µl of serum- free medium and starved for an hour at 37 °C. After starvation, cells were treated with different concentrations of the compound DMEHE (6- 500 µg/ml) with three replicates of each concentration for 24 h. At the end of the treatment period the medium was refreshed with serum- free medium containing MTT (0.5 mg/ml). The microtiter plates were incubated for four hours in dark at 37 °C. The MTT containing medium was then discarded and the cells were washed with PBS (200 µl). The formazan crystals formed in the cells were dissolved with 100 µl of DMSO and the rate of the color developed was measured. Spectrophotometric absorbance of the purple blue formazan dye was measured in a microplate reader (Bio-Rad 680, India) at 570 nm**.** IC50 values were determined by calculating the % of cell viability. Percentage of cell viability was calculated by using formula: % viability= (live cell count/ total cell count)* 100. Data are reported as mean IC50 values± S.D from three independent experiments. The graph was plotted with cell viability against various concentrations of the compound.

## Results

**Structural elucidation of the compound**

Based on the spectral data, the structure of the compound was identified as -2-benzene dicarboxylic acid, mono 2-ethylhexyl ester (DMEHE) and the molecular formula was determined as C_16_H_22_O_4_. UV- visible absorption spectra of DMEHE in methyl alcohol showed λ max of 222, FT-IR (cm^-1^) spectra at 3130,1404 corresponds to C-C, 2875.86, 858-704 corresponds to C-H, 1298, 1124 corresponds to C-N and 948 indicates the presence of O- H groups in the compound. The ^1^H- NMR spectrum (CDCl_3_; 400 MHz) was obtained with 0.8- 0.85 (m, 6H, CH3), 1.2- 1.4 (m, 9H, CH2, CH), 3.8 (d, 2H, CH2) and 7.56- 7.74 (m, Ar- 4H). The ^13^C-NMR spectrum was observed with 170.1 (COOH), 168.6 (C=O), 133.8 (Ar-c), 132.9 (Ar-c), 132.8 (Ar-c), 132.5 (Ar-c), 128.4 (Ar-c), 128.0 (Ar-c), 66.9 (CH2), 38.9 (CH), 31.9 (CH2), 29.3 (CH2), 23.7 (CH2), 23.0 (CH2), 14.1(CH3) and 11.6 (CH3). The 3D–structures of the compound (Figure 2) was modelled using Chem3D Ultra software (Version 8). The compound is white in color, soluble in methanol, ethyl acetate and DMSO. The physico- chemical properties of DMEHE are given in table 1.

**Fig. 1:**
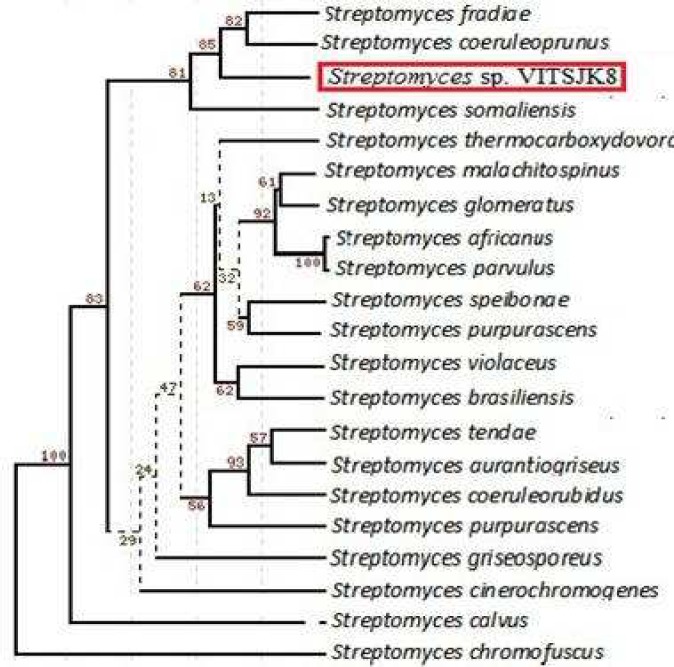
Phylogenetic tree of *Streptomyces* sp. VITSJK8 based on 16S rDNA. Score bar represents one nucleotide substitution per 100 nucleotides.

**Fig. 2:**
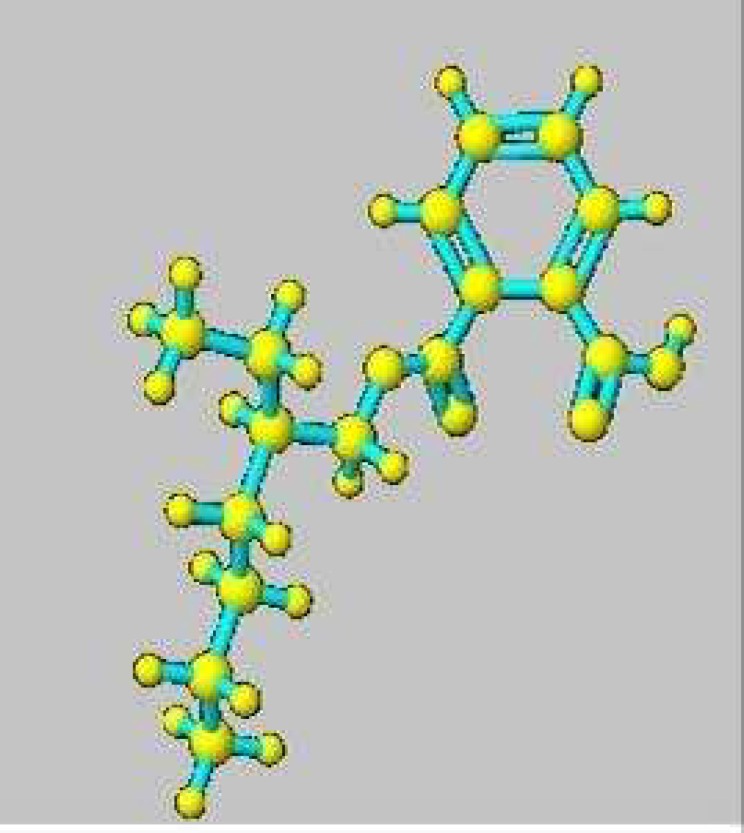
3D structure of 1, 2-benzene dicarboxylic acid, mono 2-ethylhexyl ester (DMEHE) modelled in Chem3D Ultra 8.0

**Table 1:** Physicochemical properties of DMEHE

Average mass: 278.343506 Da	Molar Volume: 255.8±3.0 cm3
Monoisotopic mass: 278.151794 Da	Polar surface area 63.6A
Boiling Point: 408.9±28.0 °C at 760 mmHg	Molar Refractivity: 77.3±0.3 cm3
Vapour Pressure: 0.0±1.0 mmHg at 25°C	Enthalpy of Vaporization: 69.7±3.0 kJ/mol
Density: 1.1±0.1 g/cm3	Surface Tension: 41.7±3.0 dyne/cm
Enthalpy of Vaporization: 69.7±3.0 kJ/mol	Flash Point: 144.1±17.5 °C
Polarizability: 30.7±0.5 10-24cm3	H bond acceptors:4
Freely Rotating Bonds:9	H bond donors:1
Average mass: 278.343506 Da	Molar Volume: 255.8±3.0 cm3
Monoisotopic mass: 278.151794 Da	Polar surface area 63.6A**˚**
Boiling Point: 408.9±28.0 °C at 760 mmHg	Molar Refractivity: 77.3±0.3 cm3
Vapour Pressure: 0.0±1.0 mmHg at 25°C	Enthalpy of Vaporization: 69.7±3.0 kJ/mol
Density: 1.1±0.1 g/cm3	Surface Tension: 41.7±3.0 dyne/cm
Enthalpy of Vaporization: 69.7±3.0 kJ/mol	Flash Point: 144.1±17.5 °C
Polarizability: 30.7±0.5 10-24cm3	H bond acceptors:4
Freely Rotating Bonds: 9	H bond donors:1

**Cytotoxic activity of** **1, 2-Benzene dicarboxylic acid, mono (2-ethylhexyl) ester**

**Fig. 3:**
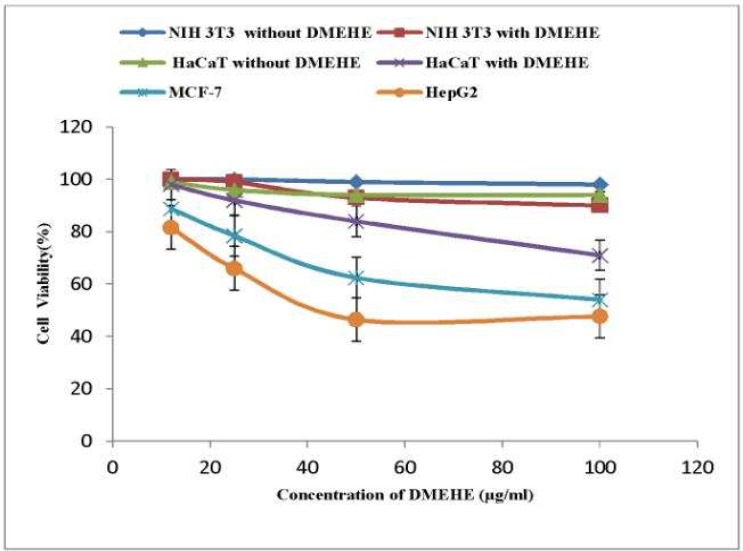
Cytotoxic activity of different concentrations of DMEHE on various cell lines. Values are mean of three independent experiments; standard error bars are shown

*In vitro* cytotoxicity assays of bioactive lead compound (DMEHE) extracted from *Streptomyces *sp. VITSJK8, against the cell lines revealed that DMEHE exhibited cytotoxic activities on HepG2 and MCF-7 cancer cell lines and it showed less cytotoxic activity on normal cell lines, NIH 3T3 and HaCaT by MTT assay in 96 well plates. The MTT assay is commonly used to determine mitochondrial reductive function and hence, is a good indicator of cell death or inhibition of growth**. **The different concentrations of DMEHE tested against normal cell lines exhibited less toxicity with IC50> 500 µg/ ml against NIH 3T3 and IC50> 250 µg/ ml against HaCaT cell line. DMEHE exhibited a cytotoxic activity on cancer cell lines. Among the cell lines, HepG2 was more susceptible and the cell viability was decreased to 68%, followed by MCF-7 (78%), HaCaT (83%) and NIH 3T3 cells (96%) by MTT assay. The results showed that the DMEHE exhibited cytotoxicity on all the cancerous cells in a concentration- dependent manner. Each cell line was treated with increasing concentrations (6- 250 µg/ ml) of DMEHE for 24 h. Untreated cells were used as a negative control. HepG2 was found to be more susceptible followed by MCF-7, HaCaT and NIH 3T3 cells (Figure 3).

**Fig. 4:**
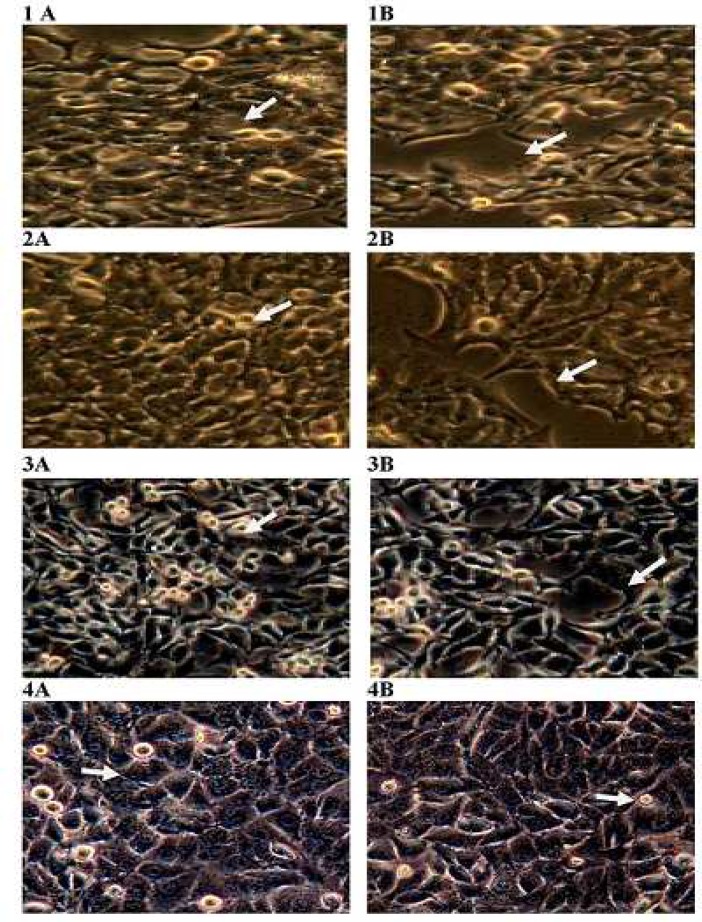
Morphological alteration of different cell lines. Pannel A: untreated NIH 3T3 (1), HaCaT (2), MCF-7 (3) and HepG2 (4) cell lines. Pannel B: 100 µg/ ml DMEHE treated NIH 3T3 (1), HaCaT (2), MCF-7 (3) and HepG2 (4) cell lines Arrows indicate morphological changes observed.

The morphological changes observed in various cancer cells after treatment with DMEHE are shown in figure 4. Morphological alteration of HepG2, MCF-7, NIH3T3 and HaCaT cell lines after the exposure of DMEHE at various concentrations were observed under phase contrast microscope. Cells exposed to 50 µg/ ml and higher concentrations of DMEHE for 24 h showed morphological changes when compared to control cells. In all cancer cell line, morphological changes which include cell rounding up, cell adhesion capacity, apoptotic feature with membrane blebbing were observed.

## Discussion

Marine actinobacteria *Streptomyces* sp. VITSJK8 was isolated from a sediment sample collected at Cheyyur beach, Bay of Bengal, Kanchipuram, India. Large scale fermentation was carried out to obtain sufficient quantity of crude extract for the purification and identification of the bioactive compound. The culture broth yielded a 150.3 mg of pure compound. The purified compound was subjected to UV, FT-IR, ^1^H NMR and ^13^CNMR spectroscopic studies in order to establish the chemical structure of the bioactive compound. The 3D- structures of the pure compound was established from the spectral data.

In this study, the bioactive actinomyceteal metabolite DMEHE extracted from *Streptomyces* sp. VITSJK8 was evaluated for cytotoxic activity on four different cell lines (HepG2, MCF- 7, NIH 3T3 and HaCaT). DMEHE showed potential cytotoxic activity against HepG2 and MCF- 7 cell lines. It showed less cytotoxic activity on HaCaT and NIH 3T3 normal cell lines. The observed IC50 values suggest that DMEHE was less toxic to normal cell lines (NIH3T 3 and HaCaT) when compared to the malignant cell lines (HepG 2 and MCF- 7). *Streptomyces* sp. is considered as potentially important source of novel bioactive anticancer compounds and capable of producing chemically diverse compounds without any side effects for variety of clinical applications (19). The antifungal compound 5-(2, 4- dimethylbenzy l) pyrrolidin- 2- one (DMBPO) extracted from marine *Streptomyces *VITSVK5 spp. exhibited cytotoxic activity on HEP 2 and Hep G2 cell lines with IC50 values of 2.8 and 8.3 µg/ ml, respectively, as compared to Vero cell line 22.6 µg/ ml (20). Naphthomycin A, a cytotoxic compound from *Streptomyces* sp. nov. WH26 have been shown to exhibit potent cytotoxicity against several cancer cell lines. It was reported that Naphthomycin A showed IC50 values of 9.2 ± 0.8, 15.2 ± 1.1, 7.9 ± 1.4 and 20.8 ± 1.6 l M against A549, HeLa, BEL-7402 and HT-29 cell lines respectively (21). The above reports suggest that compounds extracted from *Streptomyces *showed high cytotoxic activity against cancer cell lines and less cytotoxicity towards normal cell lines.

The HepG2 cell line was found to be more sensitive to DMEHE after 24 h of incubation by MTT assay. The differences in sensitivity might be due to the characteristics of each cell line and their origin (22). The reduction in percentage of cell viability revealed that the bioactive compound DMEHE showed cytotoxic activity at different concentrations on various types of cancerous cell lines.

Cytotoxic mechanism of natural bioactive compounds, interfere with basic cellular functions such as cell cycle, apoptosis, inflammation, angiogenesis, invasion and metastasis. (23). Many anticancer drugs from marine organisms cause cytotoxicity and cell death through the induction of apoptosis (24). In the present study, morphological changes of the cells were observed at concentration- dependent manner in DMEHE treated cells. Microscopic images of DMEHE treated cancer cells revealed that cytotoxicity may be due to the induction of apoptosis evidenced by the display of apoptotic cellular morphology (25). Due to** t**he increased incidence of drug resistance by many tumor cells, there is a need to explore microbial-derived secondary metabolites as anticancer drugs with specific antitumor activity to circumvent the demand for anticancer chemo-therapeutic agents (26). Several cytotoxic comp-ounds are available in the market, however, most anticancer drugs lack tumor specificity and cause damage to normal tissues leading to side effects (27). Natural products from marine derived actinomycetes are an important resource for new metabolites. In our present study, screening of secondary metabolites obtained from *Streptomyces* sp. VITSJK8 lead to the identification of bioactive compound 1, 2- benzene dicarboxylic acid, mono 2- ethylhexyl ester which exhibited potential cytotoxic activity against HepG 2 and MCF- 7 cancer cell lines. *Streptomyces* sp.VITSJK8 can be explored further as a source of novel metabolites with cytotoxic activity.
